# Novel lncRNA LNC_000113 Drives the Activation of Pulmonary Adventitial Fibroblasts through Modulating PTEN/Akt/FoxO1 Pathway

**DOI:** 10.3390/jcdd10060262

**Published:** 2023-06-15

**Authors:** Hui Luo, Lin Zhao, Ziwei Ou, Tangzhiming Li, Yanghong Liu, Zaixin Yu

**Affiliations:** 1 Department of Cardiology, The First Hospital of Changsha (Xiangya Medical College Affiliated Changsha Hospital of Central South University), Changsha 410005, China; 2 Department of Cardiovascular Medicine, The Third Xiangya Hospital, Central South University, Changsha 410013, China; 3 Department of Cardiology, Shenzhen People’s Hospital, Shenzhen 518020, China; 4 Reproductive Medicine Centre, The Third Xiangya Hospital, Central South University, Changsha 410013, China; 5 Department of Cardiology, Xiangya Hospital, Central South University, Changsha 410008, China

**Keywords:** lncRNA, pulmonary adventitial fibroblasts, PTEN, Akt, FoxO1

## Abstract

The activation of pulmonary adventitial fibroblasts (PAFs) is one of the key components of pulmonary arterial remodelling in pulmonary arterial hypertension (PAH). Emerging evidence indicates that lncRNAs may play fibrotic roles in a range of diseases. In this present study, we identified a novel lncRNA, LNC_000113, in pulmonary adventitial fibroblasts (PAFs) and characterised its role in the Galectin-3-induced activation of PAFs in rats. Galectin-3 led to elevated expression of lncRNA LNC_000113 in PAFs. The expression of this lncRNA was primarily PAF enriched. A progressive increase in lncRNA LNC_000113 expression was observed in rats with monocrotaline (MCT)-induced PAH rats. Knockdown of lncRNA LNC_000113 cancelled the Galectin-3′s fibroproliferative effect on PAFs and prevented the transition of fibroblasts to myofibroblasts. The loss-of-function study demonstrated that lncRNA LNC_000113 activated PAFs through the PTEN/Akt/FoxO1 pathway. These results propose lncRNA LNC_000113 drives the activation of PAFs and promotes fibroblast phenotypic alterations.

## 1. Introduction

Pulmonary arterial hypertension (PAH) is a devastating disease with a poor prognosis. The key pathological feature of PAH is progressive pulmonary vascular remodelling, which leads to increased pulmonary vascular resistance. The increase in pulmonary vascular resistance elevates the right ventricular load and eventually causes right ventricular failure. Pulmonary vascular remodelling includes alteration of the adventitia, media, and intima of the pulmonary arteries. Dysfunctional proliferation and apoptosis of endothelial and smooth muscle cells, in conjunction with the infiltration of inflammatory cells, have been intensively studied in pulmonary vascular remodelling in PAH. Moreover, increasing attention has been paid to fibroblasts in adventitial remodelling in PAH [1].

The pulmonary adventitial fibroblasts (PAFs) serve as an important regulator of extracellular matrix and pulmonary vascular pathology. PAFs act as “sentinel cells”, which detect changes in the extracellular matrix and trigger signalling alterations [1,2]. PAFs closely interact with inflammation and oxidative stress, causing changes in vascular function and structure [3,4]. The abnormal activation of PAFs includes phenotypic changes in myofibroblasts and increased production of extracellular matrix proteins [5]. The mechanisms contributing to the activation of PAFs in PAH are intricate, and most of them remain to be investigated.

Galectin-3 participates in a series of pathophysiological processes such as fibrosis, inflammation, apoptosis, and angiogenesis [6]. Galectin-3 is a potent fibrogenic factor in fibroblasts [7]. Our previous studies have reported increased Galectin-3 levels in the plasma of patients with PAH. Galectin-3 is also upregulated in the adventitia of the pulmonary arteries in PAH rats. The inhibition of the Galectin-3 function reduces pulmonary artery remodelling. Exogenous Galectin-3 promotes the activation of PAFs [8,9]. Further work is needed to interpret the pro-fibrotic mechanisms of Galectin-3 in PAFs.

Long noncoding RNAs (lncRNAs) are transcripts with a length of more than 200 nucleotides but do not code for proteins. lncRNAs are involved in various biological and pathological processes, including pulmonary arterial remodelling in PAH [10]. Changes in lncRNA expression have been observed in the pulmonary vascular cells. For example, the expression of lncRNA PAXIP1 antisense RNA 1 (PAXIP1-AS1) has been found to enhance the pulmonary arteries of patients with PAH. The lncRNA PAXIP1-AS1 regulates its downstream target, paxillin, which plays a role in cell proliferation and migration, as well as focal adhesion [11]. The increased expression of lncRNA COX2 has been identified in pulmonary artery smooth muscle cells (PASMCs). The silencing of lncRNA COX2 reduced hypoxia-induced PASMC proliferation and migration [12]. These are examples of lncRNAs that have been characterised in the pulmonary arterial remodelling in PAH. Many of the lncRNA studies in this field have focused on validating the biofunctions of pre-selected lncRNAs in PAH rather than identifying novel lncRNAs involved in disease pathogenesis. Additionally, there is currently limited knowledge regarding the biofunction of lncRNAs in the pulmonary adventitia in pulmonary arterial remodelling.

This study aimed to investigate the lncRNAs involved in Galectin-3-related profibrotic biology in PAFs in pulmonary adventitial remodelling in PAH. Upregulation of the novel lncRNA LNC_000113 in activated PAFs was identified using RNAseq. The role of this lncRNA in PAFs was characterised. lncRNA LNC_000113 was regulated by Galectin-3 and was required for Galectin-3-induced activation of PAFs. Increased expression of lncRNA LNC_000113 was observed in the pulmonary adventitia of the MCT rats. This study proposed a regulatory role of lncRNA LNC_000113 in fibrotic vasculopathy in adventitial remodelling in PAH.

## 2. Materials and Methods

### 2.1. MCT-Induced PAH in Rats

The animal experiments were approved by the Animal Ethics Committee of Central South University. Male Sprague Dawley rats (150–200 g body weight) were used for establishing the PAH model after one week of adaptive feeding. Rats were grouped beforehand in a randomised manner. To induce PAH, the rats received a single injection of monocrotaline (MCT) at the dose of 60 mg/kg. A high-affinity Galectin-3 inhibitor N-acetyllactosamine (N-Lac) was given to the rats via intraperitoneal injection at the dose of 5 mg/kg every other day. The injection of N-Lac started the day after MCT injection, as previously described [8,13]. The rats were randomly assigned to 4 groups: healthy controls (*Ctrl*), healthy rats injected with the Galectin-3 inhibitor (*Ctrl + Gal3i*), MCT rats (*MCT*), and MCT rats injected with the Galectin-3 inhibitor (*MCT + Gal3i*). For placebo control purposes, the rats of the two groups without N-Lac injection (*Ctrl* and *MCT*) received injections of vehicle (saline).

### 2.2. Assessment of Hemodynamics and Right Ventricular Hypertrophy

The rats received an intraperitoneal injection of pentobarbital (60 mg/kg) for anaesthesia. Appropriate anaesthetic depth was confirmed by checking the loss of the pedal reflex. The right common jugular vein was bluntly separated. An arterial clip was used to clamp the proximal end of the vein, and the distal end was ligated. An incision was made in the vein, and a catheter was inserted. The catheter was sequentially pushed deep into the right atrium, RV, and PA. The catheter was connected to a pressure transducer, and the pressure curve was continuously recorded (PowerLab, ADInstruments). Another catheter was inserted into the carotid artery of rats to obtain systolic blood pressure. Mean pulmonary artery pressure (mPAP), right ventricular systolic pressure (RVSP), and systemic systolic blood pressure (SBP) were calculated from the hemodynamic records. After the hemodynamic experiments, the rats were sacrificed via neck dislocation. The RV was dissected from the LV and septum. The weight ratio of the RV to the LV with the septum (Fulton Index) was used as an index of RV hypertrophy.

### 2.3. Cell Culture

Primary cultures of PAFs, pulmonary artery smooth muscle cells (PASMCs), and pulmonary artery endothelial cells (PAECs) were isolated from male Sprague Dawley rats as previously described [8,14]. Briefly, segments of the pulmonary artery were obtained from fresh rat lungs. For PAFs culture, the endothelium and smooth muscle layers were removed. The remaining adventitia was minced into tiny blocks and adhered to the bottom of a cell culture flask. Tissue was cultured in Dulbecco’s Modified Eagle’s medium (DMEM) supplemented with 15% fetal bovine serum and 1% streptomycin and penicillin. PAFs grew out of the tissue after approximately 5 days. For PASMC culture, the endothelium and adventitia were carefully removed from the pulmonary artery segment. The tissue was then minced, and the tissue blocks were cultured in DMEM with 15% fetal bovine serum and 1% antibiotics (streptomycin and penicillin). PASMCs grew out from the tissue in about 7 days. For PAECs culture, the endothelium of the pulmonary artery was carefully collected. The PAECs were extracted via enzymatic digestion with Collagenase type I (Sigma-Aldrich, Burlington, DE, USA). PAECs were cultured in Dulbecco’s modified Eagle’s medium/Ham’s F-12 medium (DMEM/F12) supplemented with 15% fetal bovine serum and 1% antibiotics (streptomycin and penicillin). The cells were purified using the differential adhesion method. The 3–5 passages were used for the experiment. For Galectin-3 treatment, cells were cultured with 5 µg/mL recombinant Galectin-3 (R&D) for 24 h before processing to indicated assays.

### 2.4. Western Blotting

RIPA buffer containing protease inhibitor cocktail (Beyotime, Shanghai, China) was used to extract proteins from lung tissues or PAF cells. Protein samples were subjected to the bicinchoninic acid (BCA) assay (Beyotime, Shanghai, China) for quantification. An identical amount (20 µg) of each protein sample was loaded onto a 10% SDS-PAGE gel. The protein samples were separated on the gels and were then transferred to a PVDF membrane (Bio-Rad, Hercules, CA, USA). The membrane was blocked in 5% non-fat milk and subsequently probed with primary antibodies overnight at 4 °C. The antibodies used for Western blotting are listed in Supplementary Table S1. Membranes were then incubated with horseradish peroxidase (HRP)-conjugated goat anti-rabbit IgG. The protein blots were detected and captured using a chemiluminescence system (Bio-Rad, Hercules, CA, USA). Images were analysed using FIJI 1.53c (imagej.net). All the whole western blot figures can be found in the supplementary materials (File S1). 

### 2.5. RNA Isolation

Total RNA was isolated from cells or lung tissues using TRIzol reagent (Takara, Kusatsu, Japan), according to the manufacturer’s protocol. The absorbance at 260 nm (A260), 280 nm (A280), and 230 nm (A230) of the isolated RNA was measured using NanoDrop2000 (Thermo Fisher Scientific, Waltham, MA, USA) to assess the quality of the RNA samples. The total RNA was treated with DNase before RNA sequencing and quantitative PCR experiments.

### 2.6. RNA Sequencing

The total RNA of PAFs with or without Galectin-3 treatment was delivered to Novogene Bioinformatics (Shanghai, China) for RNA sequencing and bioinformatic analysis. Briefly, after the depletion of ribosomal RNA, sequencing libraries were generated using the rRNA-depleted RNA with the NEBNext^®^ Ultra™ Directional RNA Library Prep Kit for Illumina (NEB, Ipswich, MA, USA) following the manufacturer’s instructions. The sequencing libraries were bound to complementary adapter oligos using a TruSeq PE Cluster Kit v3-cBot-HS (Illumina, San Diego, CA, USA). The constructed libraries were sequenced, and 150 base-pair paired-end clean reads were generated using an Illumina HiSeq 4000 platform. Raw reads were obtained from the FASTQ files. The subsequent bioinformatics analysis included quality control, read alignment, transcriptome assembly, expression quantification, lncRNA screening, and differential expression analysis. The raw reads were aligned to the reference genome (UCSC rn6, RGSC Rnor_6.0) using HTSeq (0.9.1). Transcriptome assembly and quantification of the transcripts were performed using StringTie (v1.3.3). Ballgown (2.12.0) was used for differential expression analysis.

### 2.7. Quantitative Real-Time PCR (qPCR)

Isolated RNA was reverse transcribed using the RevertAid First Strand cDNA Synthesis Kit (Thermo Fisher Scientific, Waltham, MA, USA) according to the manufacturer’s protocol. Real-time PCR was performed with the cDNA template, SYBR Premix Ex Taq Kit (Takara, Kusatsu, Japan), and primer pairs using the Applied Biosystems 7300 Real-Time PCR System (Applied Biosystems, Waltham, MA, USA). The list of primers used (Sangon Biotech, Shanghai, China) is provided in Supplementary Table S2. Three or more biological replicates were used for each experiment. Each biological replicate was analysed in triplicate (technical replicates). The relative expression of the target transcripts was calculated using the comparative Ct method with normalization to GAPDH transcripts.

### 2.8. RNA Fluorescence in Situ Hybridization

RNA fluorescence in situ hybridization (FISH) was performed for in situ visualization of lncRNA LNC_000113 in paraffin-embedded lung tissues. The Cy3-labeled lncRNA LNC_000113 probe was designed and synthesised (RiboBio, Guangzhou, China) for FISH (probe sequence is listed in Supplementary Table S3). FISH assays of lung tissue were performed using a fluorescence in situ Hybridization Kit (RiboBio, Guangzhou, China) according to the manufacturer’s protocol. Following FISH, nuclei were stained with DAPI (Sigma-Aldrich). A confocal microscope (Leica, Germany) was used for image acquisition. The average fluorescence intensity of the adventitia was quantified using FIJI 1.53c (imagej.net).

### 2.9. LncRNA Knockdown with Antisense Oligonucleotide GapmeRs

Both LNC_000113 and negative antisense oligonucleotide (ASO) GapmeRs were supplied by Qiagen/Exiqon for lncRNA knockdown. The sequences of the ASO GapmeRs are shown in Supplementary Table S4. The antisense oligonucleotides GapmeRs were transiently transferred to PAFs using Lipofectamine RNAiMAX (Invitrogen, Waltham, USA), according to the manufacturer’s protocol. Knockdown efficiency was checked using qPCR, and the results are presented in Supplementary Figure S1.

### 2.10. CCK-8 Cell Viability Assay and EdU Cell Proliferation Assay

Cell viability was measured using the Cell Counting Kit-8 (Beyotime, Shanghai, China), following the manufacturer’s instructions. Briefly, cells were seeded into 96-well plates at a density of 5000 cells per well and synchronised overnight. The cells were then treated with 5 µg/mL recombinant Galectin-3 (R&D) or vehicle (PBS) for 24 h. Next, 10 µL of CCK-8 solution was added to each well and incubated for 1 h. Absorbance at 450 nm was measured using a high-sensitive Microplate Reader (Infinite F200; Tecan Corp., Männedorf, Switzerland). Cell proliferation was detected using the EdU assay (RiboBio, Guangzhou, China), according to the manufacturer’s instructions. Briefly, cells were seeded into 96-well plates at a density of 5000 cells per well and synchronised overnight. The cells were then incubated with 50 μM EdU for 2 h and fixed in 4% paraformaldehyde fixation solution. After cell fixation, the cells were stained with a 1X Apollo reaction cocktail for 30 min. The nuclei were stained with Hoechst33342. A fluorescence microscope (Leica, Wetzlar, Germany) was used for image acquisition. The percentage of the cells with EdU staining was quantified using FIJI 1.53c (imagej.net).

### 2.11. Statistical Analysis

Statistical analyses were performed using R, version 4.1.1. All values are presented as mean ± SD unless otherwise stated. One-way analysis of variance (ANOVA) was used for multi-group analysis. Multiple comparisons were performed using Tukey’s Honest Significant Difference test. *p* < 0.01 was considered as significantly different. Significance code in the figures: * for *p* < 0.05, ** for *p* < 0.01, and *** for *p* < 0.001.

## 3. Results

### 3.1. Galectin-3 Promoted the Remodeling of the Pulmonary Vascular Adventitia in PH

**Figure 1:**
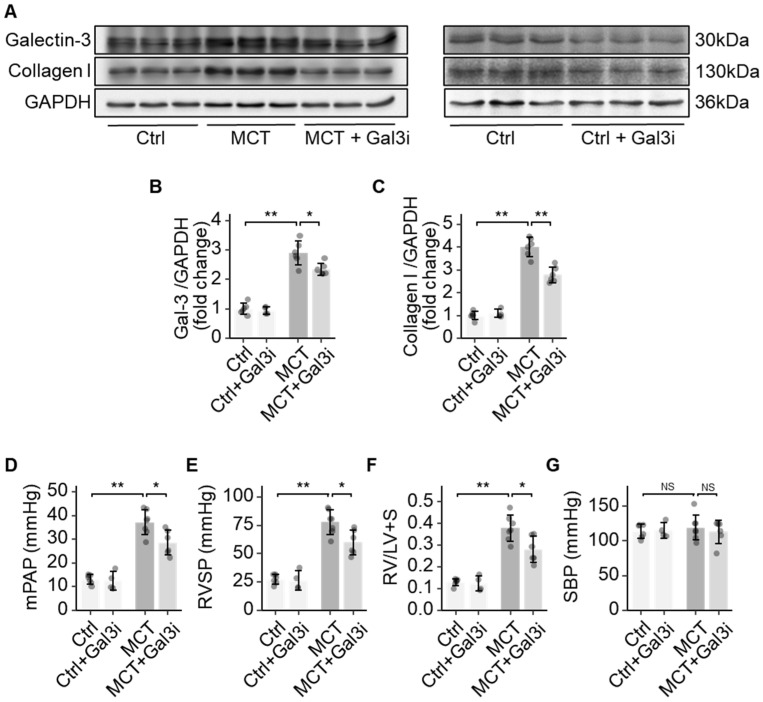
Galectin-3 promoted fibrosis in pulmonary arterial remodelling. (**A**) Representative immunoblots showing the protein expression of Galectin-3 and Collagen I in the lungs of healthy controls (*Ctrl*), control rats with Galectin-3 inhibition (*Ctrl + Gal3i*), MCT rats (*MCT*), and MCT rats with Galectin-3 inhibition (*MCT + Gal3i*); (**B**) Quantification of the protein expression of Galectin-3 in the lungs of the 4 groups; (**C**) Quantification of the protein expression of Collagen I in the lungs of the 4 groups; (**D**) Comparison of the mean pulmonary artery pressure (mPAP) in the 4 groups; (**E**) Comparison of the right ventricular systolic pressure (RVSP) in the 4 groups; (**F**) Comparison of Fulton’s Index (weight ratio of RV to LV plus septum) in the 4 groups; (**G**) Comparison of the systolic systemic blood pressure (SBP) in the 4 groups. Sample size N = 4~6 for each group. Significance code: NS stands for not significant; * for *p* < 0.05, ** for *p* < 0.01.

Galectin-3 is involved in various pathological processes such as inflammation, proliferation, and fibrosis, but its role in the vasculopathy of PAH is poorly understood [15]. Our previous studies identified increased Galectin-3 in the lungs of hypoxic rats [16]. Here, we observed increased Galectin-3, along with increased collagen I deposition in the lungs of MCT-induced PAH rats (Figure 1A–C). Pharmacologically inhibiting Galectin-3 using N-acetyllactosamine decreased the level of Galectin-3 (Figure 1B). Galectin-3 inhibition (*Gal3i*) decreased collagen I deposition in the lungs of MCT rats (Figure 1C). Reduced pressure in the pulmonary artery and the RV was observed in the MCT rats treated with the Galectin-3 inhibitor, as significant decreases in mean pulmonary arterial pressure (mPAP) and right ventricular systolic pressure (RVSP) were recorded (Figure 1D,E). Galectin-3 inhibition also attenuated hypertrophic changes in the RV (the weight ratio of RV over LV plus septum, RV/LV+S) in MCT rats (Figure 1F). Systemic blood pressure, quantified as the systolic blood pressure (SBP) of MCT rats, was not changed via Galectin-3 inhibition (Figure 1G). Galectin-3 inhibition did not alter the collagen level, the RV hypertrophy, and the haemodynamic in the healthy control rats.

### 3.2. RNA Sequencing Identified lncRNA LNC_000113 as a Galectin-3-Regulated lncRNA

**Figure 2:**
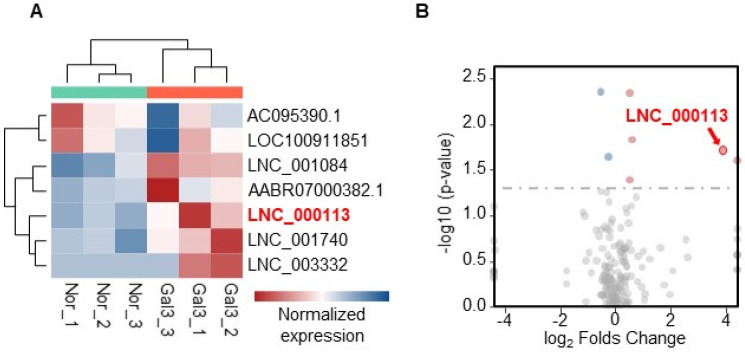
RNA sequencing identified differentially expressed lncRNAs that responded to Galectin−3 treatment. (**A**) Heatmap visualizing the expression of the 7 differentially expressed lncRNAs; (**B**) Volcano plot illustrating the expression changes of lncRNAs in PAFs with Galectin-3 treatment. lncRNA LNC_000113 was highlighted in red in the plots.

LncRNAs may participate in Galectin-3-mediated profibrotic processes. To search for potential lncRNAs, RNAseq was performed on PAFs with or without Galectin-3 treatment. RNAseq identified seven differentially expressed lncRNAs (Supplementary Table S1). A heatmap visualizing the expression levels of the seven differentially expressed lncRNAs is shown (Figure 2A). Among these seven lncRNAs, the expression of lncRNA LNC_000113 was more than 14 times higher in PAFs with Galectin-3 treatment than in the control group (adjusted *p* = 0.01938). A volcano plot demonstrating lncRNA expression in PAFs with Galectin-3 versus PAFs without Galectin-3 is presented (Figure 2B). The sequence information for lncRNA LNC_000113 is shown in Supplementary Table S2.

### 3.3. lncRNA LNC_000113 Is Highly Expressed in Galectin-3-Treated PAFs and Remodelled Pulmonary Artery Adventitia

**Figure 3:**
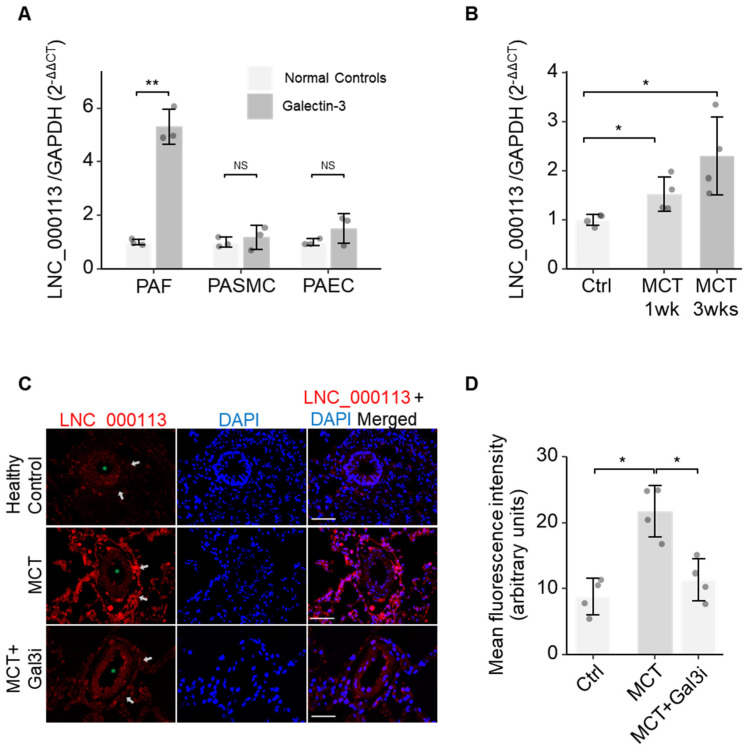
Increased expression of lncRNA LNC_000113 in PAFs and remodelled pulmonary artery adventitia. (**A**) Expression of lncRNA LNC_000113 in PAFs, PASMCs, and PAECs with Galectin−3 treatment; (**B**) Quantification of the expression of lncRNA LNC_000113 in different stages (1 week and 3 weeks) of MCT rats. (**C**) RNA fluorescence in situ hybridization visualised the localization of the primary expression of lncRNA LNC_000113 in pulmonary artery adventitia (indicated with white arrows). The green asterisk (*) sign indicates the lumen of the pulmonary arteries. Scale bar = 50 μm; (**D**) Quantification of the fluorescence intensity of lncRNA LNC_000113 in healthy controls and MCT rats; Abbreviations: PAFs, pulmonary adventitial fibroblasts; PASMCs, pulmonary artery smooth muscle cells; PAECs, pulmonary arterial endothelial cells. Fluorescent staining: DAPI (blue), lncRNA LNC_000113 (red). Sample size N = 3 for each group in cell experiments of Galectin−3 treatment. Sample size N = 4 for each group in the MCT rat experiments. Significance code: NS stands for not significant; * for *p* < 0.05 and ** for *p* < 0.01.

To validate the role of lncRNA LNC_000113 in PAH vasculopathy, we checked its expression in pulmonary arterial cells. A significant increase in lncRNA LNC_000113 expression was observed in PAFs cultured with Galectin-3 treatment. Galectin-3 did not alter the expression of lncRNA LNC_000113 in PASMCs and PAECs (Figure 3A). FISH analysis of lung tissues revealed abundant expression of lncRNA LNC_000113 in the pulmonary artery adventitia of MCT rats (Figure 3B). The expression of lncRNA LNC_000113 was significantly increased in the pulmonary artery adventitia of MCT rats compared to that in healthy control rats (Figure 3C). Increased expression of lncRNA LNC_000113 was observed in the lungs of 1-week MCT rats, and a further increase was observed in 3-week MCT rats (Figure 3D). This suggested a progressive increase in lncRNA LNC_000113 in parallel with the severity of pulmonary adventitial remodelling in PAH rats.

### 3.4. Knocking Down lncRNA LNC_000113 Prevented Galectin-3-Induced PAFs’ Activation

**Figure 4:**
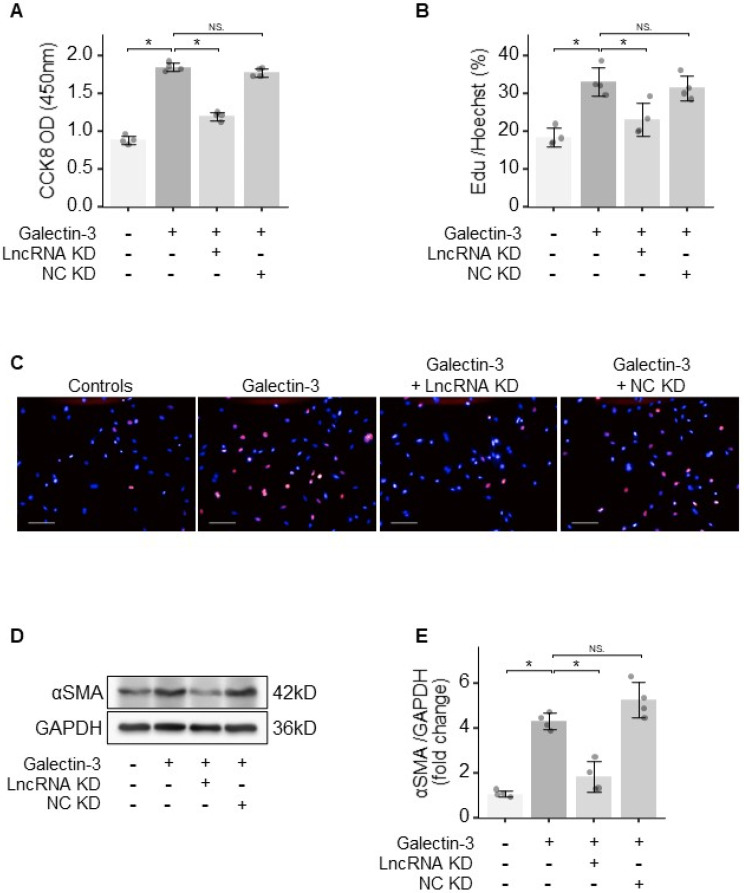
lncRNA LNC_000113 activated PAFs. (**A**) CCK−8 cell viability assay of PAFs with different interventions; (**B**) EdU cell proliferation assay of PAFs with different interventions; (**C**) Representative images of EdU staining of PAFs with different interventions; Scale bar = 100 μm; (**D**) Representative immunoblots of αSMA showing the differentiation of fibroblasts to myofibroblasts in PAFs with different interventions; (**E**) Quantification of the protein expression of αSMA in PAFs with different interventions. LncRNA KD: lncRNA LNC_000113 knockdown with antisense oligonucleotide GapmeRs; NC KD: negative control GapmeR. Sample size: N = 4 for each group. Significance code: NS stands for not significant; * for *p* < 0.05.

To characterise the contribution of lncRNA LNC_000113 in the activation of PAFs, loss-of-function experiments of this lncRNA were performed. Galectin-3 treatment increased cell viability (assessed using CCK-8) in PAFs. The knockdown of lncRNA LNC_000113 using GapmeRs cancelled the Galectin-3-induced increase in cell viability in PAFs (Figure 4A). EdU assays showed increased proliferation of PAFs following Galectin-3 treatment. The knockdown of lncRNA LNC_000113 significantly suppressed the proliferation of Galectin-3-treated PAFs (Figure 4B,C). Galectin-3 facilitated cell transition to myofibroblasts, as an increased αSMA marker was found in PAFs with Galectin-3 treatment. The knockdown of lncRNA LNC_000113 restrained the Galectin-3-induced transition to myofibroblasts in PAFs, as a reduced expression of αSMA was observed in Galectin-3-treated PAFs (Figure 4D,E). These results showed that lncRNA LNC_000113 is required for Galectin-3-induced activation of PAFs, and the knockdown of lncRNA LNC_000113 reduced Galectin-3-induced proliferation and activation in PAFs.

### 3.5. lncRNA LNC_000113 Promotes PAFs’ Activation through PTEN/Akt/FoxO1 Pathway

**Figure 5:**
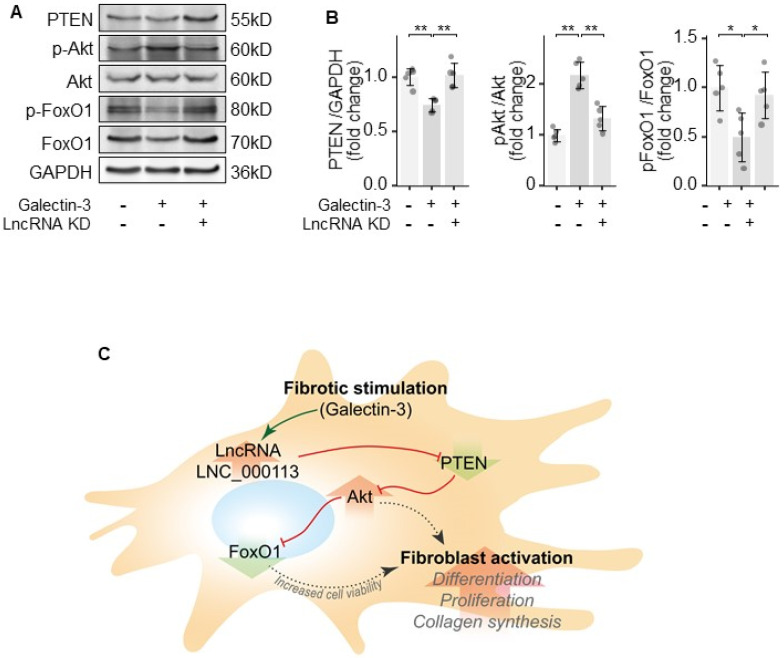
lncRNA LNC_000113 modulates PTEN/Akt/FoxO1 pathway in PAFs. (**A**) Representative immunoblots of PTEN, Akt, phosphorylated Akt, FoxO1, and phosphorylated FoxO1 in PAFs; (**B**) Quantification of the expression of PTEN, Akt, phosphorylated Akt, FoxO1, and phosphorylated FoxO1 in PAFs; (**C**) Proposed mechanism of the profibrotic effect of lncRNA LNC_000113 in PAFs. Sample size N = 5 for each group. Significance code: * for *p* < 0.05, ** for *p* < 0.01.

To figure out the regulatory network of lncRNA LNC_000113, the key molecules in the PTEN/Akt/FoxO1 pathway were examined. Galectin-3 treatment in PAFs decreased PTEN expression, increased Akt phosphorylation, and decreased the expression of FoxO1. The knockdown of lncRNA LNC_000113 reversed Galectin-3-induced changes in the PTEN/Akt/FoxO1 pathway in PAFs (Figure 5A,B). The knockdown of lncRNA LNC_000113 in PAFs treated with Galectin-3 restored PTEN expression, reduced the phosphorylation of Akt, and elevated the expression of FoxO1 (Figure 5A,B). These results suggested that lncRNA LNC_000113 could increase cell variability through the PTEN/Akt/FoxO1 pathway in PAFs, which may consequently contribute to the activation of PAFs. The proposed signalling regulation mechanism is shown in Figure 5C.

## 4. Discussion

In this study, we identified a novel lncRNA, LNC_000113, as an activator of PAFs in rats with PAH. Galectin-3 is a potent activator of pulmonary adventitial remodelling that promotes fibroproliferation in PAFs. RNA sequencing revealed a significantly higher expression of lncRNA LNC_000113 in PAFs with Galectin-3 treatment. An increased expression of this lncRNA was solely observed in Galectin-3-treated PAFs instead of in other pulmonary arterial cells. Moreover, a progressive upregulation of lncRNA LNC_000113 was observed in the lungs of the MCT rats. Loss-of-function experiments suggested that lncRNA LNC_000113 was required for Galectin-3-induced activation of PAFs. The underlying mechanism involves the modulation of the PTEN/Akt/FoxO1 pathway. These results indicated a profibrotic role of lncRNA LNC_000113 in pulmonary arterial remodelling. The manipulation of lncRNA LNC_000113 could serve as a potential therapeutic target for PAH.

Progressive pulmonary arterial remodelling is a hallmark of PAH. Pulmonary adventitial remodelling is associated with the extracellular matrix and perivascular environment and plays a role in essential vasculopathy, such as fibrosis, inflammation, and oxidative responses [3,4]. The abnormal activation of PAFs is an essential component of pulmonary adventitial remodelling [17]. The activation of PAFs includes phenotypic changes, such as transition into myofibroblasts, altered synthesis of extracellular matrix proteins, and promotion of pathological changes in other pulmonary arterial cells [3,5,8]. The mechanisms contributing to the activation of PAFs in PAH are intricate, and most of them remain to be investigated.

Our previous study proposed an important role for Galectin-3 in pulmonary arterial adventitia in PAH [8]. In this present study, the upregulation of Galectin-3 was checked in the lungs of MCT rats. Galectin-3 inhibition attenuated collagen deposition and haemodynamic deterioration in the lungs of MCT rats. These results were in line with our previous observations and those of other researchers [8,15,18]. Fibroblasts are the cells primarily responsible for collagen production and secretion. PAFs contribute to collagen deposition, as they are able to differentiate into myofibroblasts and secrete more extracellular matrix components, such as collagen, contributing to pulmonary vascular remodelling. Our previous study showed that Galectin-3-specific inhibitors or genetic deletion of Galectin-3 reversed fibrotic pulmonary arterial remodelling in MCT rats [18]. In this current study, we again observed that the Galectin-3 inhibitor prevented pulmonary vascular remodelling in MCT rats. It should be noted that few studies have reported drugs that reverse pulmonary artery remodelling in PAH. Our study used a prevention design, as it is unlikely that the galectin-3 inhibitor (N-Lac) would be able to reverse the progression of vasculopathy in PAH. The current literature support that Galectin-3 induced the proliferation, differentiation, and extracellular matrix deposition of PAFs [8,15]. Our observations supplement the understanding of the profibrotic role of Galectin-3 in perivascular remodelling in PA.

Although Galectin-3′s fibroproliferative effects have been widely reported, the regulatory mechanisms related to pulmonary adventitial remodelling and other fibrogenic processes remain largely unknown. In order to study the possible lncRNA mechanisms involved, we used RNA sequencing for high-throughput screening of the Galectin-3-regulated lncRNAs and found seven differentially expressed lncRNAs. Among them, a novel lncRNA, LNC_000113, had a significantly larger change in expression and higher statistical significance. To date, no study has referred to this lncRNA as fibrosis. The role of lncRNA LNC_000113 in PAF activation was then investigated.

This study demonstrated the profibrotic role of lncRNA LNC_000113 in PAFs. Here, we observed a close relationship between the upregulation of lncRNA LNC_000113 and the profibrotic factor Galectin-3. An increase in lncRNA LNC_000113 was also observed in the remodelled pulmonary adventitia of PAH rats. Studies have reported the role of lncRNA in fibrotic pathogenesis [19]. Some of these claims an anti-fibrotic role of the lncRNA [20,21]. For example, lncRNA maternally expressed gene 3 (MEG3), an intensively studied anti-fibrotic lncRNA, prevents the production of matrix metalloproteinase-2 and reduces cardiac fibrosis through a p53-dependent mechanism. LncRNA MEG3 reduced collagen formation in pulmonary fibrosis by suppressing the transforming growth factor-β1 and PI3K/AKT pathway [21]. LncRNAs involve in the activation and transition of fibroblasts into myofibroblasts as well [22]. Rudi et al. reported the profibrotic mechanism of fibroblast-enriched lncRNA WISPER in the myocardium. The lncRNA WISPER specifically targets cardiac fibroblasts and controls a large range of pathological processes, including fibroblast differentiation, proliferation, apoptosis, and migration [19]. lncRNA PVT1 promoted the activation, proliferation, and migration of fibroblast cell lines by suppressing the antifibrotic effect of miR-497-5p [22]. The expression of lncRNA PVT1 can be enhanced by FOXM1, a profibrotic factor, leading to excessive pulmonary fibrosis [22]. The lncRNA ZFAS1 was increased in the lungs of rats with pulmonary fibrosis induced by TGF-β1. It facilitated the transition of fibroblasts to myofibroblasts by regulating miR-150-5p/SLC38A1 [23]. Currently, there is limited knowledge regarding the biofunctions of lncRNA LNC_000113. In our study, lncRNA LNC_000113 was identified as a key regulator of fibrosis in the remodelled pulmonary adventitia. A limitation of our study was that lncRNA LNC_000113 knockdown was not performed in vivo in PAH rats. In the future, more comprehensive experiments are needed to validate the profibrotic effects of this lncRNA in PAH rodents.

Although knowledge regarding lncRNA participation in pulmonary adventitial remodelling is limited, an increasing number of studies have linked lncRNAs with pulmonary arterial remodelling [10]. For example, lncRNA H19 was increased in the serum and lung of MCT-induced PH in rats and mice [24]. LncRNA H19 was induced by PDGF, interleukins 1β, and interleukins 6 in PASMCs, promoting PASMC proliferation via regulating Angiotensin II receptor type 1 [24,25]. Increased lncRNA TYKRIL expression was identified in hypoxic PASMCs and pericytes from patients with idiopathic PAH. LncRNA TYKRIL promoted proliferation and suppressed apoptosis in PASMC and pericytes by regulating the p53/PDGF signalling [26]. Additionally, increased lncRNA SMLIR expression was observed in hypoxic PASMCs, PAH patients, and PAH experimental rodents. LncRNA SMILR facilitated pulmonary arterial remodelling by promoting RhoA/ROCK signalling [27]. LncRNA PAHRF acted as a competing endogenous RNA in PASMCs. LncRNA PAHRF regulates serine/threonine kinase 4 by sponging microRNA-23a-3p, regulating cell proliferation and apoptosis [27].

We further figured out the profibrotic mechanism of lncRNA LNC_000113 through the PTEN/Akt/FoxO1 pathway. PTEN is a lipid phosphatase that suppresses Akt activation. PTEN has been reported to be a negative regulator of fibroblast activation in rodent models of bleomycin- or lipopolysaccharide-induced pulmonary fibrosis, and the deletion of PTEN magnified the effects of fibrosis [28,29]. Additionally, PTEN upregulation relieved pulmonary fibrosis and reduced fibroblast activation and collagen secretion by suppressing Akt activity [29]. FoxO1 is a downstream target of Akt, which is negatively regulated by Akt-mediated phosphorylation [30]. Phosphorylated FoxO1 translocates from the nucleus to the cytoplasm, resulting in reduced transcriptional activity. FoxO1 suppression facilitates fibrogenic processes [31]. The deactivation of FoxO1 was linked to bleomycin-induced pulmonary fibrosis [32]. Our study provides evidence for the involvement of the PTEN/Akt/FoxO1 pathway in the activation of PAFs. These results suggested that lncRNA LNC_000113 is a regulator of the PTEN/Akt/FoxO1 pathway. The interplay between this lncRNA and the key molecules in this pathway could be further explored. For example, lncRNA-related competitive endogenous RNA networks have been extensively studied recently. LncRNA LNC_000113 may serve as a competitive endogenous RNA that inhibits key molecules such as PTEN and leads to downstream signalling changes.

As currently no literature focused on lncRNAs in pulmonary adventitial remodelling, our study provides preliminary insights into the role of a novel lncRNA in the biology of adventitial PAFs. This study demonstrated that lncRNA LNC_000113 was highly abundant in activated PAFs and remodelled pulmonary adventitia. This lncRNA was required for Galectin-3-induced activation of PAFs, in terms of cell viability, proliferation, and myofibroblast differentiation. Further investigations linked the profibrotic effect of lncRNA LNC_000113 with the regulation of the PTEN/Akt/FoxO1 pathway. The manipulation of this lncRNA could serve as a target for anti-fibrotic therapeutics in pulmonary arterial remodelling.

## Data Availability

The data that support the findings of this study are available from the corresponding author upon reasonable request.

## Supplementary Materials

The following supporting information can be downloaded at: https://www.mdpi.com/article/10.3390/jcdd10060262/s1, Table S1. Antibodies used in the experiment; Table S2. Primers for qRT-PCR; Table S3. Sequences of probes for FISH; Table S4. Sequences of targeting and control ASO GapmeRs; Table S5. Commercial kits used in the study; Table S6. Information of the differentially expressed lncRNAs; Table S7. Sequence of lncRNA LNC_000113; Figure S1. Knockdown efficiency of ASO GapmeRs.
