# Supplementary material for: Novel lncRNA LNC_000113 Drives the Activation of Pulmonary Adventitial Fibroblasts through Modulating PTEN/Akt/FoxO1 Pathway
Source: J Cardiovasc Dev Dis. 2023 Jun 15;10(6):262. doi: 10.3390/jcdd10060262 (PMC10299350; doi:10.3390/jcdd10060262)

Original blots for Figure 1

Galectin 3

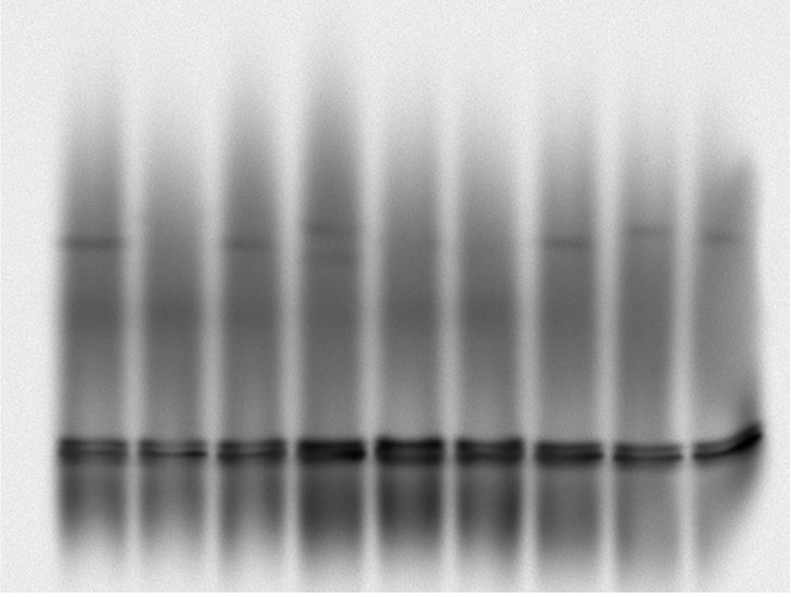

Collagen I

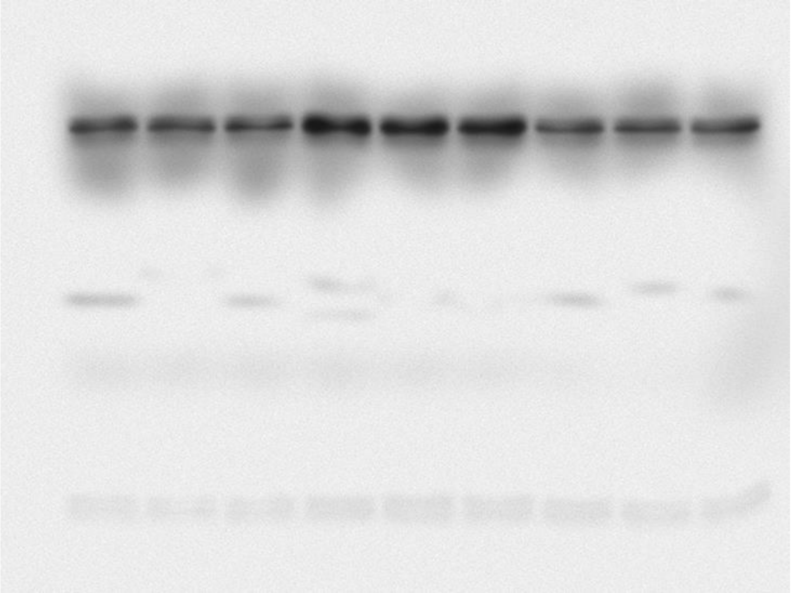

GAPDH

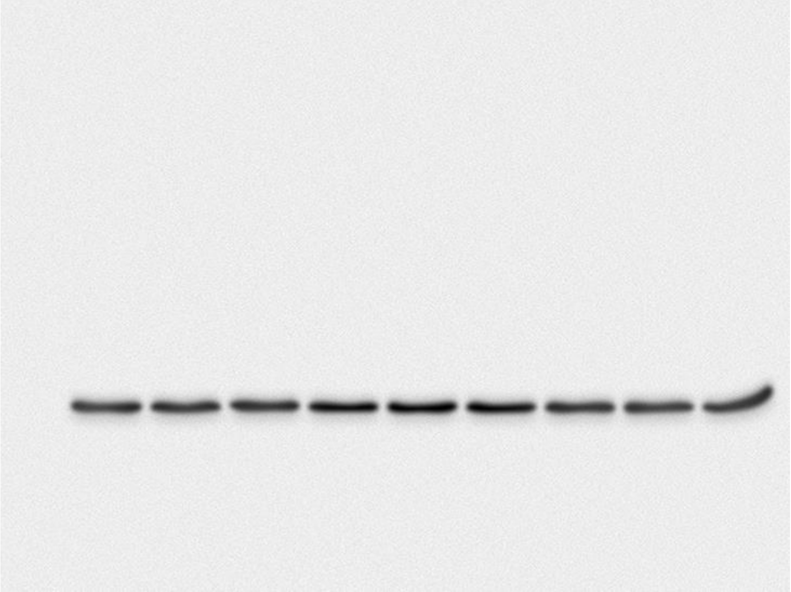

Original blots for
Figure 4

SMA

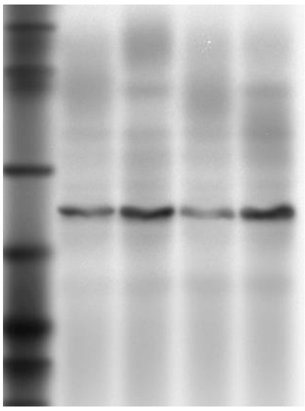

GAPDH

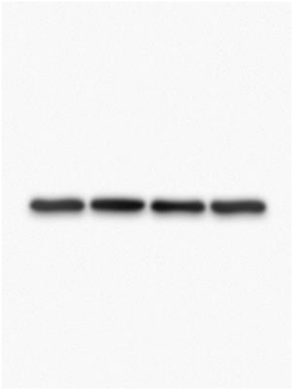

Original blots for Figure 4

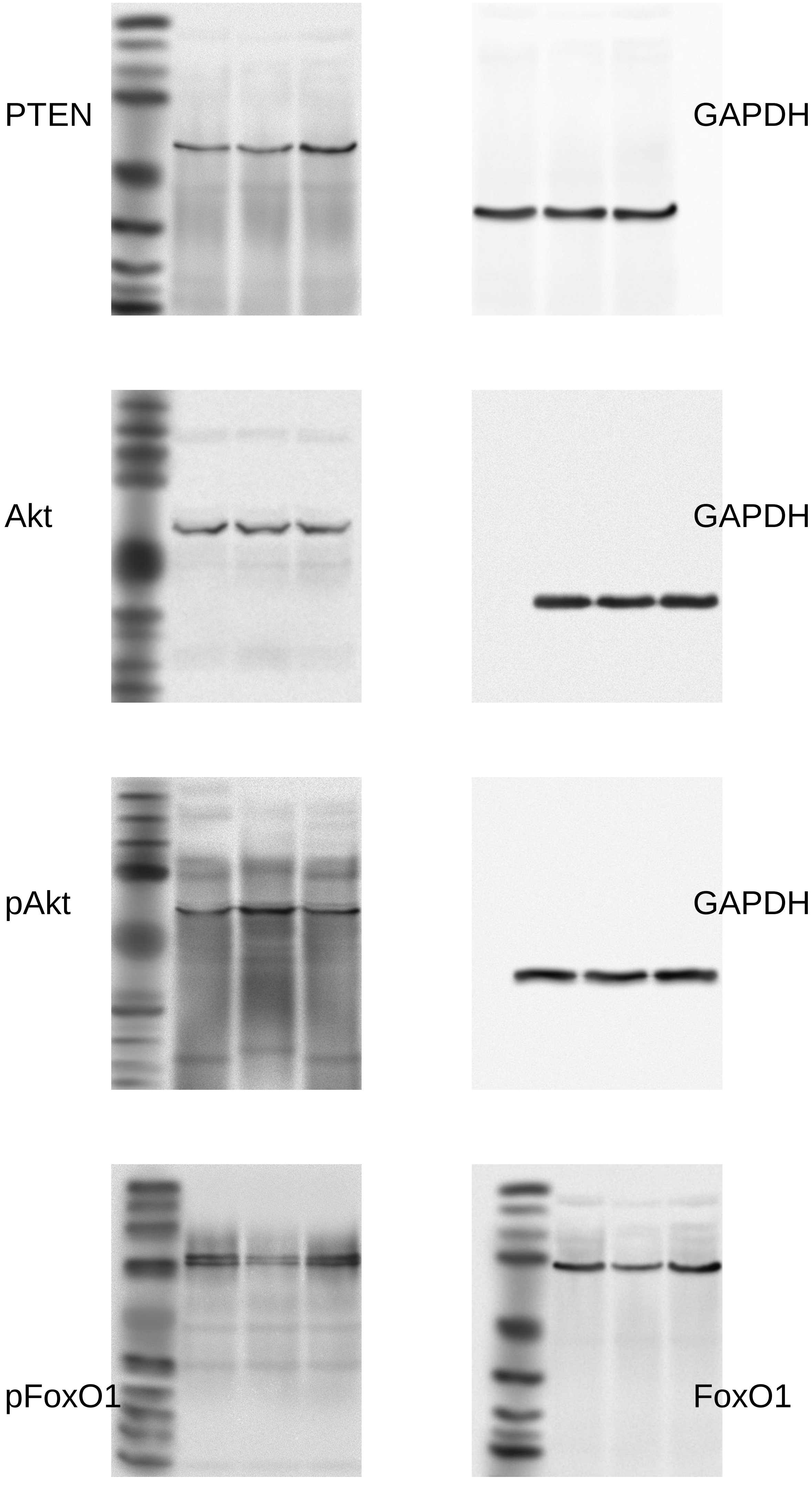

Supplement: Supplementary file 1 [file jcdd-10-00262-s001.zip › jcdd-2286001-supplementary/File S1 whole western blots.pdf]
