# Supplementary material for: Novel lncRNA LNC_000113 Drives the Activation of Pulmonary Adventitial Fibroblasts through Modulating PTEN/Akt/FoxO1 Pathway
Source: J Cardiovasc Dev Dis. 2023 Jun 15;10(6):262. doi: 10.3390/jcdd10060262 (PMC10299350; doi:10.3390/jcdd10060262)

Supplementary Material

Table S1. Antibodies used in the experiment.

Antibody targets	Supplier	Catalog. No.	Host	Applications	Molecular weight (kDa)
Galectin-3	Proteintech	14979-1-AP	Rabbit	WB (1:1000)	31
Collagen I	Proteintech	14695-1-AP	Rabbit	WB (1:1000)	130
α-SMA	Proteintech	14395-1-AP	Rabbit	WB (1:1000)	45
PTEN	Proteintech	22034-1-AP	Rabbit	WB (1:1000)	55
Akt	Cell Signaling	9272S	Rabbit	WB (1:1000)	60
p-Akt	Cell Signaling	9271S	Rabbit	WB (1:1000)	60
FoxO1	Cell Signaling	18592-1-AP	Rabbit	WB (1:2000)	70
p-FoxO1	Cell Signaling	9461S	Rabbit	WB (1:1000)	80
GAPDH	Proteintech	10494-1-AP	Rabbit	WB (1:5000)	37

Table S2. Primers for qRT-PCR

Targets	Primer	Sequence(5′-3′)
LNC_000113	forward	TCCCAATAGACGGCATAGTA
	reverse	ACCATGATACTTAGATTCCG
GAPDH	forward	TCTCTGCTCCTCCCTGTTCT
	reverse	ATCCGTTCACACCGACCTTC

Table S3. Sequences of probes for FISH

5’-cy3-CCATTCTTCAAAACTCCACTTACTACTATGCCGTCTA-cy3-3’

Table S4. Sequences of targeting and control ASO GapmeRs

Sequence Description	Oligonucleotide Sequence (5'-3')*
ASO targeting rat LNC_000113 (KO1)	TAGTTCCCAGTATCAG
ASO targeting rat LNC_000113 (KO2)	CGAATCTCAGTAACAT
Random sequence control ASO (NC KO)	TGGGCGTATAGACGTG

*The ASO GapmeRs were fully phosphorothioated.

Table S5. Commercial kits used in the study

Kits name	Supplier	Catalog. No.
NEBNext® Ultra™ Directional RNA Library Prep Kit for Illumina	NEB, USA	E7420L
TruSeq PE Cluster Kit v3-cBot-HS	Illumia	PE-401-3001
RevertAid First Strand cDNA Synthesis Kit	Thermo Fisher Scientific	K1622
SYBR Premix Ex Taq Kit	Proteintech	RR420
Fluorescence in situ Hybridization Kit	RiboBio	C10910
Lipofectamine RNAiMAX	Invitrogen	13778150
Cell Counting Kit-8	Beyotime	C0039
EdU assay Kit	Beyotime	C0071S
BCA assay Kit	Beyotime	P0011

Table S6. Information of the differentially expressed lncRNAs

Transcript ID	Ensembl Gene ID	lncRNA name	LncRNA Type	Chromosome	Start	End	Strand	Exon number	Length	log_2 (fold change)	p value
ENSRNOT00000077618	ENSRNOG00000052761	AC095390.1	Annotated	12	51760127	51840207	-	4	732	-0.52	0.004459
ENSRNOT00000081586	ENSRNOG00000060990	AABR07000382.1	Annotated	1	11766992	11772215	+	6	1158	0.51	0.004592
LNC_001084	-	-	Novel	15	51464542	51465202	+	2	510	0.60	0.015006
**LNC_000113**	**-**	**-**	**Novel**	**1**	**180643784**	**180700261**	**+**	**3**	**501**	**3.87**	**0.019381**
ENSRNOT00000087733	ENSRNOG00000061155	LOC100911851	Annotated	11	27566879	27577691	+	5	2437	-0.26	0.022833
LNC_003332	-	-	Novel	9	29458774	29526925	-	2	228	inf	0.024961
LNC_001740	-	-	Novel	2	18576098	18587270	-	2	402	0.53	0.041201

Table S7. Sequence of lncRNA LNC_000113

chr1: 180643784-180700261

TGAGCCGAATCTGAGTAACATGGTGAATTCGGTTAGTTCCCAATAGACGGCATAGTAGTAAGTGGAGTTTTGAAGAATGGAATCTAAGTATCATG
GTGAATTCAGTAGGTTCCCTATAGGAATCGCATGTAATAAGCTTCTTATTCAATATAGAGAAACGTTTTAACGAACTGAAACACTGTTTCTTTGTG
AATTCAGTTAGTTCCCAGTATCAGACTCTTGTAATATGTGTTGTTTTAAAGATTTATCAAAATTTCCTGAGCCGAATCTCAGTAACATGTTGAATTC
GGTTAGTTACCAATAGGCCACAGTAGTAATAAGTGGACTCTTCGGGATATAGAAAAATTTTCACGAATGGAATCAAAATTTCAGGGCGAATTCAG
TGAGTTCCCAATGGGACATGATTGTCTGAGTGTGCTTTTCAATACAGAGCAACATTTTAAGGAACGGAATCTAAGTATCATGGTGAATTCATAGG
ATCCCAATAGGATTCGCATGTAA

Figure S1. Knockdown efficiency of ASO GapmeRs

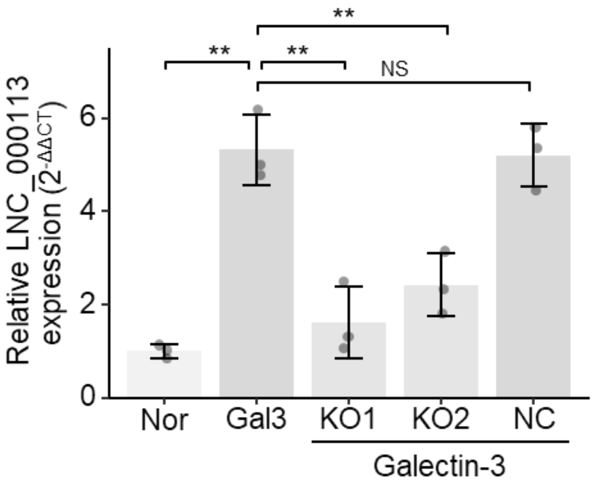

Supplement: Supplementary file 1 [file jcdd-10-00262-s001.zip › jcdd-2286001-supplementary/jcdd-2286001-supplementary.pdf]
